# Continuous low-intensity ultrasound attenuates IL-6 and TNFα-induced catabolic effects and repairs chondral fissures in bovine osteochondral explants

**DOI:** 10.1186/s12891-019-2566-4

**Published:** 2019-05-04

**Authors:** Neety Sahu, Hendrik J. Viljoen, Anuradha Subramanian

**Affiliations:** 1 Department of Chemical and Biomolecular Engineering, University of Nebraska-Lincoln, Lincoln, NE 68588-0643 USA; 2 Department of Chemical and Materials Engineering, University of Alabama at Huntsville, Huntsville, Alabama 35899 USA

**Keywords:** Inflammation, Cytokines, Ultrasound, Cartilage, Repair

## Abstract

**Background:**

Cartilage repair outcomes are compromised in a pro-inflammatory environment; therefore, the mitigation of pro-inflammatory responses is beneficial. Treatment with continuous low-intensity ultrasound (cLIUS) at the resonant frequency of 5 MHz is proposed for the repair of chondral fissures under pro-inflammatory conditions.

**Methods:**

Bovine osteochondral explants, concentrically incised to create chondral fissures, were maintained under cLIUS (14 kPa (5 MHz, 2.5 Vpp), 20 min, 4 times/day) for a period of 28 days in the presence or absence of cytokines, interleukin-6 (IL-6) or tumor necrosis factor (TNF)α. Outcome assessments included histological and immunohistochemical staining of the explants; and the expression of catabolic and anabolic genes by qRT-PCR in bovine chondrocytes. Cell migration was assessed by scratch assays, and by visualizing migrating cells into the hydrogel core of cartilage-hydrogel constructs.

**Results:**

Both in the presence and absence of cytokines, higher percent apposition along with closure of fissures were noted in cLIUS-stimulated explants as compared to non-cLIUS-stimulated explants on day 14. On day 28, the percent apposition was not significantly different between unstimulated and cLIUS-stimulated explants exposed to cytokines. As compared to non-cLIUS-stimulated controls, on day 28, cLIUS preserved the distribution of proteoglycans and collagen II in explants despite exposure to cytokines. cLIUS enhanced the cell migration irrespective of cytokine treatment. IL-6 or TNFα-induced increases in MMP13 and ADAMTS4 gene expression was rescued by cLIUS stimulation in chondrocytes. Under cLIUS, TNFα-induced increase in NF-κB expression was suppressed, and the expression of collagen II and TIMP1 genes were upregulated.

**Conclusion:**

cLIUS repaired chondral fissures, and elicited pro-anabolic and anti-catabolic effects, thus demonstrating the potential of cLIUS in improving cartilage repair outcomes.

**Electronic supplementary material:**

The online version of this article (10.1186/s12891-019-2566-4) contains supplementary material, which is available to authorized users.

## Background

Repair of damaged cartilage remains a biomedical burden as cartilage repair techniques such as microfracture, autologous chondrocyte implantation (ACI) and grafting methods including tissue-engineered approaches have failed to generate functional hyaline cartilage [1–3]. In addition to other factors that influence in-vivo repair outcomes, initial stages of repair typically take place in pro-inflammatory environment. A pro-inflammatory environment compromises the repair efforts by promoting cartilage degeneration [4–6]. Consequently, the efficacy of current and upcoming regenerative strategies is predicated on the mitigation of the detrimental effects of a pro-inflammatory joint environment to improve cartilage repair outcomes.

In an important study, the cytokine profile in patients with symptomatic cartilage defects as well as osteoarthritis (OA) revealed elevated levels of catabolic cytokines, including interleukin (IL), − 6 and tumor necrosis factor (TNF) α, in the synovial fluid and cartilage tissue extracts [7]. IL-6 and TNFα were also elevated in both acute and chronic phases of other joint injuries [7, 8]. IL-6 or TNFα-induced synthesis of matrix-degrading enzymes, such as metallopeptidases (MMPs) and A Disintegrin and Metalloproteinase with Thrombospondin Motifs (ADAMTS) aggrecanases, has been strongly implicated in cartilage degradation and OA pathogenesis [9]. Therefore, chondroprotective approaches that mitigate the catabolic effects of the pro-inflammatory joint environment are of interest.

Although the administration of non-steroidal anti-inflammatory drugs (NSAIDs) are the logical first-line treatment in an inflamed joint, low intraarticular residence time and side-effects including gastrointestinal ulcers and cardiotoxicity necessitate alternative therapeutic efforts [10]. Recently the chondroprotective effects of pLIUS against a potent catabolic cytokine, IL-1β on intact cartilage explants was demonstrated in vitro [11]. However, to our knowledge, the chondroprotective effects of LIUS on other catabolic cytokines that are elevated in injured cartilage and endogenously produced by the injured cartilage itself (IL-6 and TNFα) remains uninvestigated. Further, the repair of chondral fissures under cLIUS in the presence of catabolic cytokines has not been investigated. Therefore, the current study investigated the ability of continuous LIUS (cLIUS) to promote the repair of chondral fissures in the presence of pro-inflammatory cytokines, IL-6 and TNFα, shown to be elevated in injured cartilage and sustained for weeks post-injury [7].

Our work has shown that at any given frequency, cLIUS couples more energy than pLIUS [12] and that the bio-effects of cLIUS are frequency dependent [12–14]. Hence, differently from previous studies that employ pLIUS (1.5 MHz), our approach employed cLIUS at the cell resonant frequency of 5 MHz where the cLIUS-induced bio-effects were reportedly maximized [13, 14]. Further, cLIUS at 5 MHz was shown to close chondral fissures by promoting anabolic response as evidenced by a 3.88-fold higher interfacial strength and the enhancement of the cartilage phenotype when compared to controls in an in-vitro model of integration in osteochondral explant (data under peer-review). To extend our promising findings to experimental conditions that mimic a pro-inflammatory environment, the efficacy of cLIUS at 5 MHz in narrowing cartilage fissures while preserving the cartilage phenotype in the presence of IL-6 or TNFα was evaluated. Our study was predicated on the hypothesis that cLIUS enhances the cartilage phenotype, cellular migration and inhibits the expression of catabolic markers by suppressing NF-κB expression and upregulating the expression of anabolic markers. Thus, bovine osteochondral explants with cylindrical incisions simulating fissures in the cartilage were cultured in a cLIUS-assisted bioreactor developed at the University of Nebraska-Lincoln (UNL) [15] in the presence or absence of IL-6 or TNFα for a period of 28 days. Outcome analyses included histological and immunohistochemical assessments of the explants coupled with gene expression of catabolic and anabolic markers.

## Methods

### Creation of chondral fissures in the osteochondral explants and their culture

**Fig. 1:**
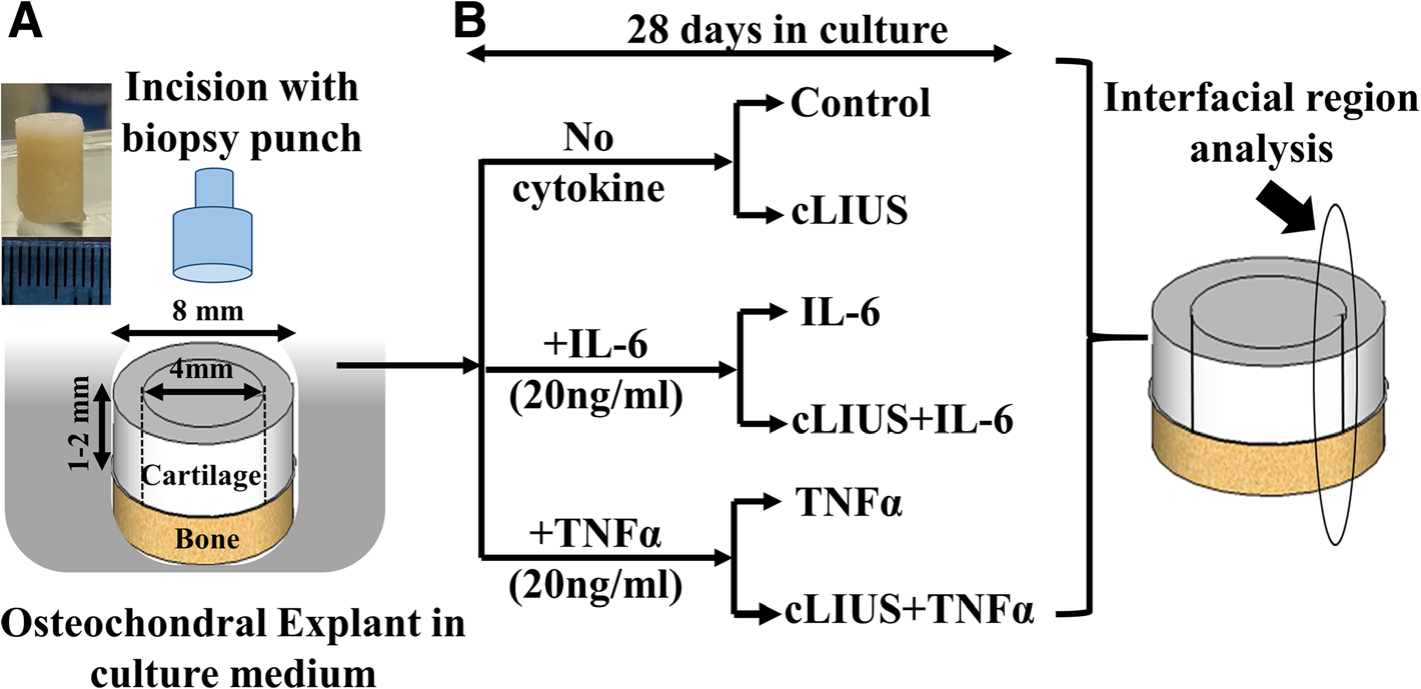
Schematic representation of the repair of chondral fissures by cLIUS under catabolic conditions: (**a**) Figure represents a schematic model of chondral fissures in bovine osteochondral explants. A 4 mm full-thickness concentric incision in the cartilage portion of the osteochondral explants (8 mm diameter, 1-2 mm chondral thickness, 1-5 mm bone segment) was created using a biopsy punch. **b** Experiment design: The incised osteochondral explants were cultured in DMEM-F12 medium supplemented with 10% FBS, 1× antibiotic-antimycotic solution and 50 μg/ml L-ascorbic acid without any cytokine treatment or in the presence of either IL-6 or TNFα, each added at a concentration of 20 ng/ml. cLIUS was applied at 14 kPa (5 MHz, 2.5 Vpp), 20 min, 4times/day for 28 days. Non-cLIUS-stimulated explants served as controls for each treatment condition

Bovine osteochondral explants (8 mm diameter, 1-2 mm cartilage thickness and 5 mm bone segment) from stifle joints were purchased from Articular Engineering (Northbrook, IL, USA). A full-thickness cylindrical incision in the cartilage segment of the osteochondral explant was created by a 4 mm biopsy punch (Integra™ Miltex®, USA). Following incision, the osteochondral explants were categorized as (a) Control (No cytokine or no-cLIUS stimulation) (b) cLIUS (c) IL-6 (d) cLIUS+IL-6 (e) TNFα (f) cLIUS+ TNFα with each study group consisting of at least 12–20 explants. The study design is schematically depicted in Fig. 1. The culture media for explants was DMEM-F12 supplemented with 10% FBS, 1× antibiotic-antimycotic solution (Gibco, USA) and 50 μg/ml ascorbic acid. IL-6 or TNFα (R&D Systems, Minneapolis, MN) was added to appropriate wells at 20 ng/ml concentration as IL-6 or TNFα dosage of 10–25 ng/ml demonstrated enhanced catabolic effects in chondrocyte cultures [16, 17]. All explants were placed in 6-well ultra-low attachment tissue culture plates (TCPs) and were either incubated in a CO_2_ incubator (37 °C, 5% CO_2_) or were cultured in the ultrasound-assisted bioreactor developed at UNL for 28 days. Media were replenished every 2 days. Sampling for outcome analyses was done on day 14 and day 28 of culture.

### Bovine articular chondrocyte culture

Bovine articular chondrocytes were isolated from stifle joints as described elsewhere [18] and cultured in DMEM-F12 supplemented with 10% FBS, 1× antibiotic-antimycotic solution (Gibco, USA) and 50 μg/ml ascorbic acid in 6 well TCPs for quantitative real time polymerase chain reaction (qRT-PCR) and scratch assays. The wells (*n* = 3 per group) were categorized as (a) Control (No cytokine or cLIUS treatment) (b) cLIUS (c) IL-6 (d) cLIUS+IL-6 (e) TNFα (f) cLIUS+ TNFα and incubated in a CO_2_ incubator (37 °C, 5% CO_2_). IL-6 or TNFα (20 ng/ml) was added to appropriate wells.

### cLIUS treatment

cLIUS (< 20 mW cm^− 2^) treatment was conducted in a custom-designed ultrasound-assisted bioreactor developed and characterized at the Department of Chemical and Biomolecular Engineering, UNL, USA, with operating procedures described elsewhere [15, 19]. cLIUS was applied to explants at 5 MHz at a constant pressure amplitude of 14 kPa at input voltage of 2.5 Vpp (peak-to-peak voltage) for 20 min per application and 4 applications per day for a period of 28 days. In a separate experiment, explants were exposed to cLIUS at 2 MHz, a frequency outside the resonant bandwidth [13], for a period of 14 days (*n* = 6) in the cLIUS-assisted bioreactor at the constant pressure amplitude of 14 kPa (6.0 Vpp, 20 min per application,4 applications/day). For adherent chondrocyte cultures, cLIUS was applied using non-focused immersion transducers (Panametrics V300, 12.7 mm diameters, Panametrix, Waltham, MA, USA) at 5 MHz and 2.5 Vpp (or 14 kPa) for either 5 or 20 min.

### Cell viability assay

Cell viability was assessed by Live/Dead Viability/Cytotoxicity kit (Molecular Probes, USA) in the osteochondral explants of each group (*n* = 3 per group per time point) on day 3 and 21 of culture. The explants were incubated with 6 μM calcein^AM^ and 4 μM ethidium homodimer-1 solution for 1 h at 37 °C under aseptic conditions and visualized by confocal microscopy (Olympus 1× 81) at 10× magnification (z step size = 5 μm, 30–40 slices per sample, ~ 150-200 μm deep) from the top or superficial zone of cartilage at the interfacial region.

### Histology and immunohistochemistry

Explants of each group (*n* = 6 per group) were typically fixed in 10% neutral buffered formalin and decalcified using a 1:1 ratio of 8% hydrochloric acid and 8% formic acid solution. The explants were cut in half and each half was paraffin embedded, sectioned and stained at the Tissue Science Facility, University of Nebraska Medical Center (Omaha, NE, USA) using standard protocol and were reviewed by Dr. Steven H. Hinrichs (Professor, Department of pathology and microbiology, UNMC, Omaha, NE, USA). Histological staining with Alcian Blue (pH 1) and Safranin O solution (Millipore, USA), and immunohistochemical staining with collagen II (ab34712, Abcam, USA) were performed on deparaffinized sections (4 μm) and visualized at 2× and 20× magnification. Normal bone tissue sections served as negative control and normal colon tissue served as positive control for collagen II staining. Fast green (Millipore, USA) was used as a counterstain for Safranin O staining. All sections were stained using the same staining solution and at the same time. Representative image of 18–36 stained interfacial regions obtained from each group (*n* = 6) was used.

### Quantification of the interfacial apposition

Alcian blue stained explants were processed to quantify percent apposition by measuring the length of the bonded interfaces (no gaps) divided by the total length of the interface [20]. Apposition was defined as the region along the interface that was bonded and did not show any discernable gaps [21]. All measurements were performed using histological images at 2× magnification (*n* = 6) using ImageJ software.

### Quantitative real-time PCR

Bovine articular chondrocytes were plated at an initial seeding density of 2 × 10^5^ cells/well. cLIUS was applied to appropriate wells for 5 min followed by homogenization with Trizol. RNA was then extracted using RNeasy Mini Kit (Qiagen, USA) as per the manufacturer’s protocol. Homogenates from 2 wells per group served as one replicate and 3 such replicates were used for analysis (*n* = 3). The qRT-PCR analysis was carried out on Realplex™ real-time PCR system (Eppendorf, USA) using TaqMan® RNA-to-CT™ 1-Step Kit (Life Technologies) as per manufacturer’s guidelines. TaqMan® probes and primers (Life Technologies, USA) used are as follows: GAPDH (Bt03210917_g1), MMP13 (Bt03214050_m1), ADAMTS4 (Bt03224697_m1), NF-κB (Bt03243457_m1), Collagen II (Bt03251861_m1) and TIMP1 (Bt03223720_m1). The expression of mRNA transcripts was normalized to GAPDH expression and relative expression levels were calculated using the 2^-ΔΔCt^ method.

### Scratch assay

Bovine articular chondrocytes were plated at a seeding density of 1 × 10^5^ cells per well (*n* = 3). Upon 90% confluency followed by serum starvation, a scratch representing a gap was created by a 200 μl pipette tip [11]. cLIUS was applied for 20 min to appropriate wells. Gap area was imaged at 0 h, 6 h, 12 h, 24 h, 48 h and 72 h following the creation of scratch by bright field inverted microscope at 5× magnification and the gap area covered by migrating cells was quantified by ImageJ.

### Migration in cartilage-hydrogel constructs

To visualize the migration of cells in a 3D format, a 4 mm central chondral region was cored out of the osteochondral explant using a biopsy punch and filled with sterile 1% agarose hydrogel. The cartilage-hydrogel constructs were categorized as (a) Control (No cytokine or cLIUS treatment) (b) cLIUS (c) IL-6 (d) cLIUS+IL-6 (e) TNFα (f) cLIUS+ TNFα and cultured in DMEM-F12 supplemented with 10% FBS, 1× antibiotic-antimycotic solution and 50 μg/ml ascorbic acid. IL-6 or TNFα was added at a concentration of 20 ng/ml and cLIUS was applied at 14 kPa (5 MHz, 2.5 Vpp) for 20 min to appropriate wells. The explants (*n* = 3 per group) were incubated with 6 μM calcein^AM^ and 4 μM ethidium homodimer-1 solution (Live/Dead Viability/Cytotoxicity kit, Molecular Probes, USA) for 1 h at 37 °C on day 24 of culture. The central region of interest was imaged by confocal microscopy (Olympus 1× 81) at 10× magnification (5 μm Z-size, 30–40 slices). Transmitted light micrograph at 10× magnification was included to distinguish hydrogel region of the construct from the native cartilage.

### Statistical analysis

The data are represented as mean ± 95% confidence interval where mean is calculated from 3 separate experiments, each conducted in triplicate per time point per study group. For gene expression and percent apposition, the statistical significance between the means of two groups with unequal variance was calculated by Welch’s test. For NF-κB gene expression, Welch’s analysis of variance (ANOVA) was used to compare control, TNFα and cLIUS+TNFα groups followed by post-hoc Games-Howell test for pair-wise comparisons. For scratch assays, two-way ANOVA with post-hoc Turkey’s test was used, and for cases where variance homogeneity was violated, Welch’s ANOVA followed by Games-Howell test was used for pair-wise comparisons. *P* values < 0.05 were considered significant and exact *p* values were indicated in the figures.

## Results

To understand the reparative and chondroprotective effects offered by cLIUS, the current study investigated the repair of chondral fissures in bovine osteochondral explants in the presence of catabolic cytokines IL-6 or TNF-α as shown schematically in Fig. 1.

### Cell viability around the chondral fissures

**Fig. 2:**
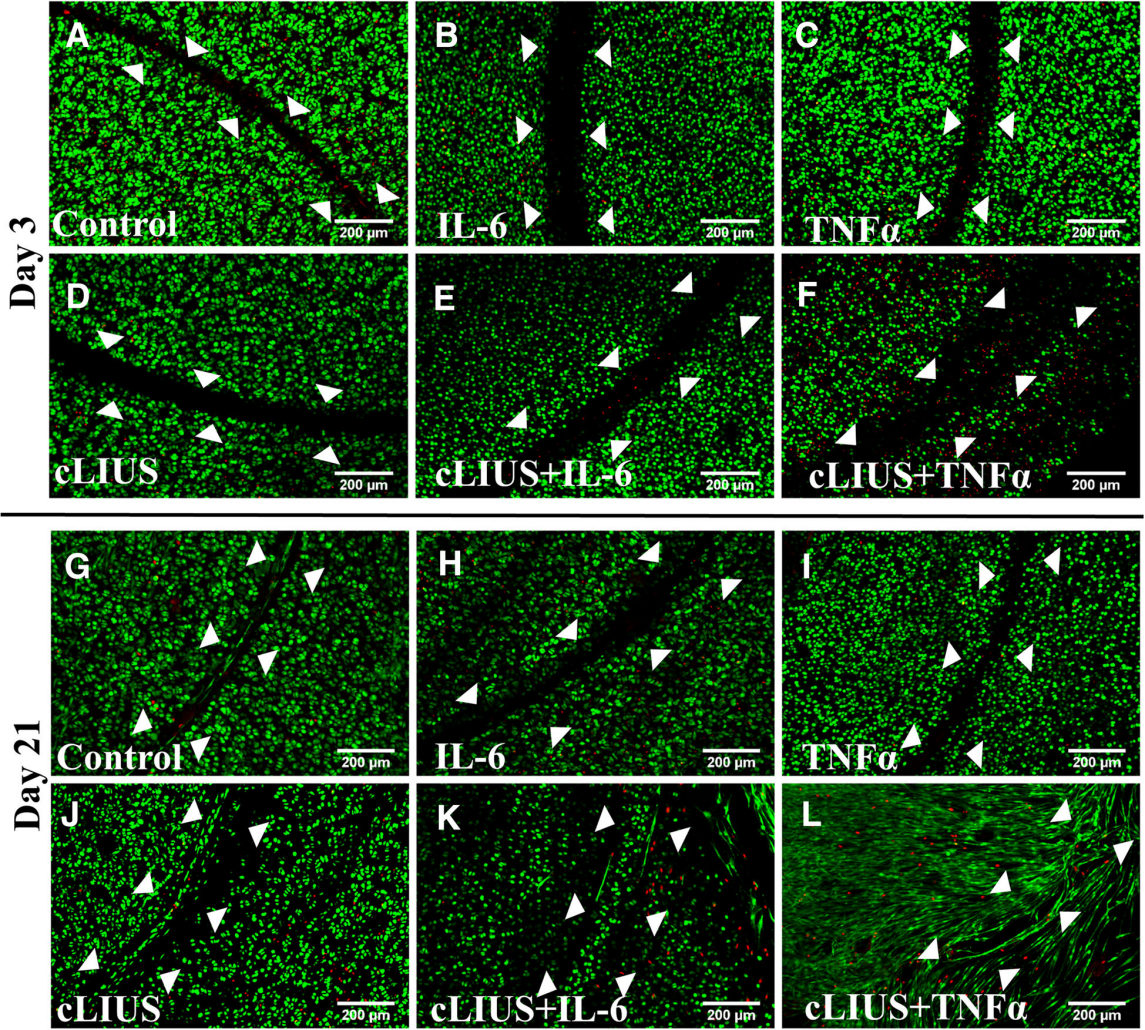
Cell viability of explants. The viability of cells at the cartilage interfacial region at the vicinity of the chondral incision in osteochondral explants was viewed by live-dead staining on day 3 and day 21 of culture. Explants (*n* = 3 per group) were incubated in 6 μM calcein^AM^ and 4 μM ethidium homodimer-1 solution for 1 h at 37 °C under aseptic conditions. Confocal micrographs viewed from the top or superficial zone of cartilage depict live (green) cells and dead (red) cells at 10× magnification. White arrowheads indicate the leading edge of the chondral incision. Scale bar represents 200 μm

Cellular viability of the osteochondral explants in the vicinity of the incision was examined by live-dead assay and shown in Fig. 2. Cells surrounding the site of incision were predominantly live (green) interspersed with few dead (red) cells. On day 3 of culture, chondral fissure was noticeable as a distinct gap in all the explants (Fig. 2a-f). After 21 days in culture, explants that received cLIUS stimulation either in the presence or absence of IL-6 or TNFα displayed enhanced cellular infiltration into the fissure when compared to non-cLIUS-stimulated controls (Fig. 2j-l).

### Repair of chondral fissures under cLIUS

**Fig. 3:**
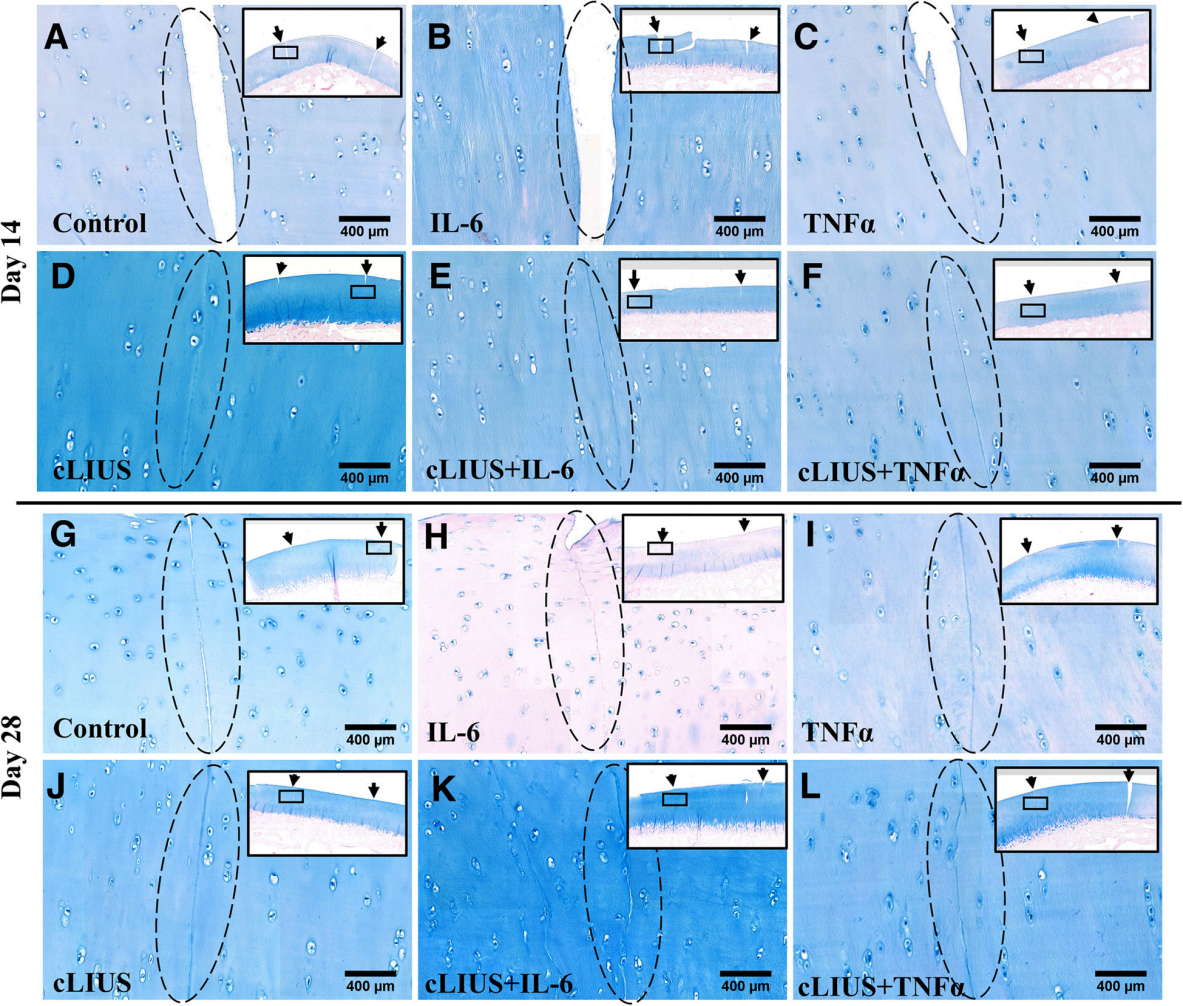
Alcian blue staining of osteochondral explants. Incised osteochondral explants (*n* = 6 explants per group per time point) were grown in DMEM-F12 medium supplemented with 10% FBS, 1× antibiotic-antimycotic solution and 50 μg/ml L-ascorbic acid in the presence or absence of IL-6 or TNFα under cLIUS at 14 kPa (5 MHz, 2.5 Vpp), 20 min, 4 times/day. Non-cLIUS-stimulated explants (*n* = 6 explants per group per time point) served as controls for each treatment condition. Explants were fixed in 10% neutral buffered formalin and embedded in paraffin. The panel shows 4 μm sections of osteochondral explants at the interfacial region stained with alcian blue (pH 1) after 14 and 28 days in culture at 20× magnification. Scale bar represents 400 μm. Insets depict the whole section imaged at 2× magnification. Black arrows in the insets depict the sites of incision

**Fig. 4:**
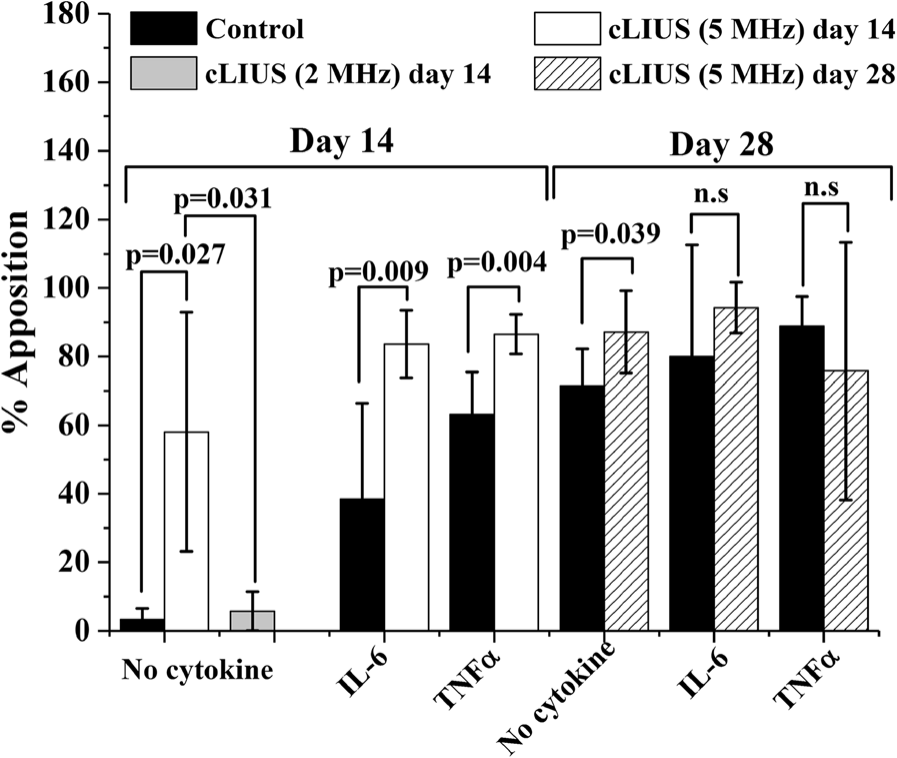
Percent apposition under cLIUS The length of chondral interfaces in the cartilage and osteochondral explants (*n* = 6 per sample per group per time point) were measured by ImageJ and the percent apposition (closed gaps) was calculated as follows: (length of closed interface/total length of interface) *100. Graph represents the percent apposition in non-cLIUS-treated and cLIUS-stimulated explants in the presence or absence of IL-6 or TNFα in osteochondral explants on day 14 and 28 of culture. Data represents a mean ± standard deviation. Statistically significant differences are indicated by *p*-values and non-significant differences are indicated as n.s

Explants were stained with alcian blue and shown in Fig. 3. Explants that received cLIUS at 5 MHz displayed closed fissures (Fig. 3d) on day 14 and a percent apposition of 58.04% (Fig. 4) was noted. In comparison, explants that received cLIUS at the non-resonant frequency of 2 MHz displayed intact gaps (Additional file 1: Figure S1) along with a percent apposition of 5.75% (Fig. 4). Percent apposition in non-cLIUS-stimulated explants at the end of 14 days was 3.32% (Fig. 4). Thus, subsequent experiments examining the effect of cytokine on the closure of fissures was performed at cLIUS at 5 MHz.

Explants that received cLIUS (5 MHz) in the presence or absence of IL-6 or TNFα displayed closed gaps on day 14 (Fig. 3d-f) and day 28 of culture (Fig. 3j-l). In contrast, non-cLIUS-stimulated explants and explants treated with either IL-6 or TNFα alone displayed intact (Fig. 3a, b) or partially narrowing fissures (Fig. 3c) on day 14, and closed fissures on day 28 of culture (Fig. 3g-i). The quantification of percent apposition corroborated the alcian blue staining results and are shown in Fig. 4. On day 14 in the absence of cytokine, the percent apposition was significantly (*p* = 0.027) increased from 3.32% in non-cLIUS-stimulated control to 58.04% under cLIUS. The inclusion of cytokines in the culture medium led to a higher percent apposition in non-cLIUS-stimulated controls. The percent apposition remained significantly elevated under cLIUS when compared to non-cLIUS-stimulated controls in the presence of IL-6 or TNFα. On day 28 in the absence of cytokine, the percent apposition was significantly higher in cLIUS-stimulated explants (*p* = 0.039) when compared to non-cLIUS-stimulated control. In contrast, no significant difference was noted between cLIUS and non-cLIUS-stimulated explants in the presence of either IL-6 or TNFα on day 28, corresponding to closed fissures observed in Fig. 3 (h, i, k, l).

**Fig. 5:**
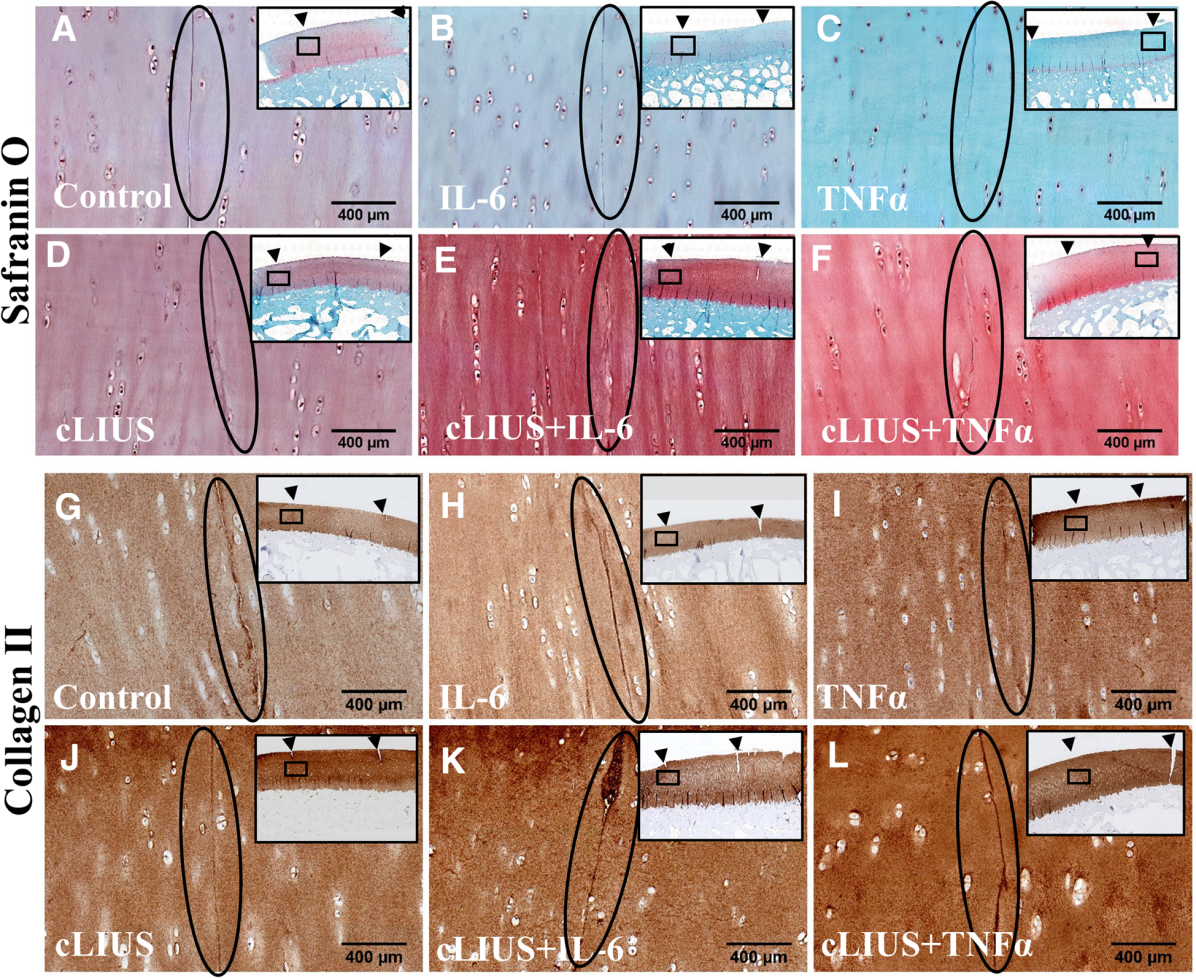
Safranin O and collagen II staining of osteochondral explants. Incised osteochondral explants cultured for 28 days under cLIUS at 14 kPa (5 MHz, 2.5 Vpp), 20 min, 4 times/day in the presence or absence of IL-6 or TNFα (*n* = 6 explants per group). Non-cLIUS-stimulated explants served as controls (*n* = 6 explants per group). Explants fixed in 10% neutral buffered formalin were paraffin embedded. The panel shows 4 μm sections of osteochondral explants at the interfacial region stained with safranin O (**a-f**) and collagen II (**g-l**) at 20× magnification. Scale bar represents 400 μm. Insets depict the whole section imaged at 2× magnification. Black arrows in the insets depict the sites of incision

After 28 days, explants exposed to IL-6 alone displayed weak alcian blue stain (Fig. 3h), indicating depletion of proteoglycans. In contrast, intense alcian blue stain was noted in IL-6-treated explants that received cLIUS stimulation (Fig. 3k). Further analysis by safranin O staining displayed noticeable depletion of proteoglycans in explants treated with either IL-6 or TNFα alone (Fig. 5b-c). cLIUS stimulation of IL-6 or TNFα-treated explants showed an intense and homogenous Safranin O stain (Fig. 5e-f). Similarly, after 28 days in culture, immunohistochemical staining of explants for collagen II exhibited weaker staining in non-cLIUS stimulated explants in the presence or absence of IL-6 or TNFα (Fig. 5g-i) when compared to cLIUS-stimulated explants (Fig. 5j-l).

Taken together, the results indicated that cLIUS at 5 MHz closed chondral fissures while preserving the proteoglycan and collagen distribution in the presence of IL-6 or TNFα; thus, demonstrating a chondroprotective effect.

### Gene expression profile under cLIUS

**Fig. 6:**
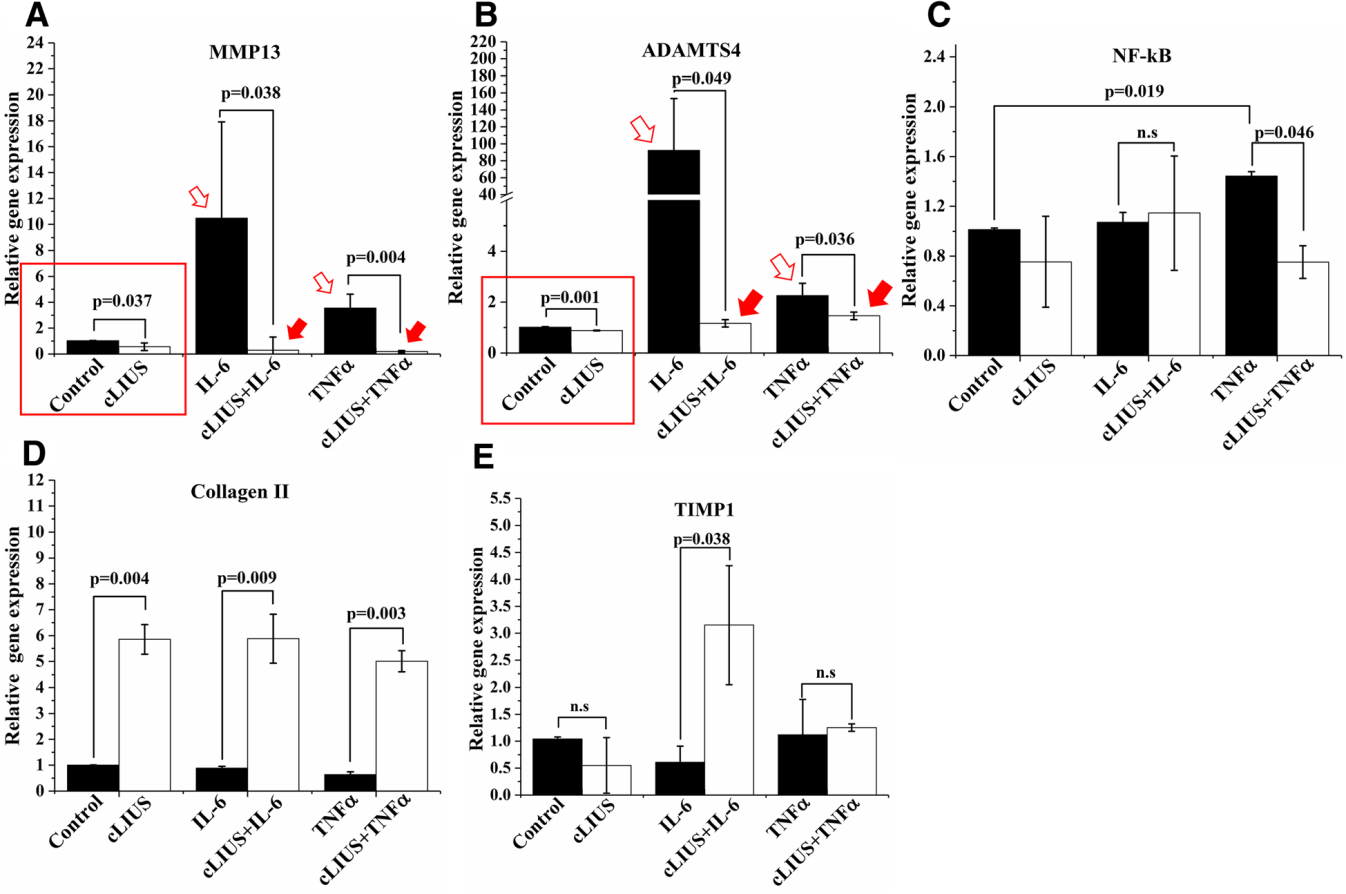
qRT-PCR analysis of the catabolic and anabolic gene expression in chondrocytes. Bovine articular chondrocytes cultured in DMEM-F12 medium supplemented with 10% FBS, 1× antibiotic-antimycotic solution and 50 μg/ml L-ascorbic acid at an initial seeding density of 2 × 10^5^ cells/well were subjected to cytokine treatment (*n* = 3) followed by cLIUS stimulation at 14 kPa (5 MHz, 2.5 Vpp) for 5 min. Non-cLIUS-stimulated explants served as controls (*n* = 3). Homogenates from 2 wells per group served as one replicate and 3 such replicates were used for analysis (*n* = 3). Total RNA was extracted and the gene expression of (**a**) MMP13, (**b**) ADAMTS4, (**c**) NF-κB, (**d**) Collagen II and (**e**) TIMP1 was evaluated by qRT-PCR. Bar graph represents mean ± 95% confidence interval, *p* values indicate statistically significant differences and n.s represents non-significant differences

To further ascertain the chondroprotective effect of cLIUS, the expression of select catabolic (MMP13, ADAMTS4) and anabolic genes (Collagen II and TIMP1) were evaluated in cultured chondrocytes by qRT-PCR and shown in Fig. 6. The expression of the transcription factor (NF-κB) in regulating the expression of catabolic genes was also assayed. In the absence of any cytokine insult, the exposure of chondrocytes to cLIUS reduced the gene expression of MMP13 (*p* = 0.037) and ADAMTS4 (*p* = 0.001) when compared to non-cLIUS-stimulated controls (indicated by rectangle in Fig. 6a, b). The inclusion of IL-6 or TNFα significantly elevated the gene expression of MMP13 (10.48 ± 7.43-fold in IL-6 and 3.55 ± 1.07-fold in TNFα) and ADAMTS4 (92.26 ± 61.10-fold in IL-6; 2.26 ± 0.46-fold in TNFα) in non-cLIUS-stimulated chondrocytes (indicated by open arrows in Fig. 6a, b). Whereas, cLIUS diminished the IL-6 or TNFα-induced upregulation of the catabolic markers (*p* = 0.038 in cLIUS+IL-6 versus IL-6, and *p* = 0.004 in cLIUS+TNFα versus TNFα for MMP13; *p* = 0.049 in cLIUS+IL-6 versus IL-6, and *p* = 0.036 in cLIUS+TNFα versus TNFα for ADAMTS4), and returned the gene expression to baseline levels observed under cLIUS-stimulation only (indicated by closed arrows in Fig. 6a, b). As anticipated, the exposure of chondrocytes to TNFα led to an increase in the gene expression of NF-κB (1.44 ± 0.03-fold in TNFα versus 1.01 ± 0.01-fold in control, *p* = 0.019) while no discernable differences in NF- κB gene expression were noted when chondrocytes were exposed to IL-6 (Fig. 6c). Under cLIUS, the gene expression of NF- κB was suppressed in chondrocytes exposed to TNFα (0.75 ± 0.13-fold in cLIUS+ TNFα versus 1.44 ± 0.03-fold in TNFα, *p* = 0.046) (Fig. 6c).

The gene expression of the cartilage-specific marker, collagen II, remained significantly elevated (5.85 ± 0.57-fold *p* = 0.004 versus non-cLIUS-stimulated control) under cLIUS stimulation irrespective of IL-6 (5.88 ± 0.95-fold in cLIUS+IL-6 versus 0.89 ± 0.07-fold in IL-6; *p* = 0.009) or TNFα treatment (5.01 ± 0.41-fold in cLIUS+TNFα versus 0.63 ± 0.11-fold in TNFα; *p* = 0.003) (Fig. 6d). Significant increases (3.15 ± 1.10-fold in cLIUS+TNFα versus 0.61 ± 0.30-fold in TNFα, p = 0.038) in the gene expression of TIMP1, an anabolic inhibitor of metalloproteinases, was observed when IL-6-treated chondrocytes were exposed to cLIUS (Fig. 6e). TNFα treatment had no significant effect on the gene expression of TIMP1 with or without cLIUS stimulation.

The results indicated that the catabolic genes were downregulated under cLIUS and this downregulation was sustained in the presence of pro-inflammatory cytokines IL-6 and TNFα. In addition, cLIUS stimulation demonstrated elevated levels of anabolic genes regardless of cytokine treatment.

### Migration of cells under cLIUS

**Fig. 7:**
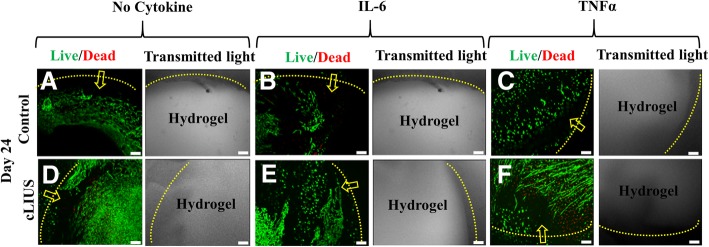
Cell migration to the hydrogel core from the surrounding cartilage in cartilage-hydrogel constructs. A 4 mm chondral core in osteochondral explants was excised using a biopsy punch and filled with cell-free 1% agarose hydrogel to form cartilage-hydrogel constructs and cultured in DMEM-F12 medium supplemented with 10% FBS, 1× antibiotic-antimycotic solution and 50 μg/ml L-ascorbic acid for 24 days in the presence or absence of IL-6 or TNFα. Constructs (*n* = 3) were exposed to cLIUS at 14 kPa (5 MHz, 2.5 Vpp), 20 min, 4 times/day (**d-f**). Non-cLIUS-stimulated constructs (*n* = 3) served as controls (**a-c**). Confocal micrographs demonstrate infiltrated cells from the surrounding cartilage into the hydrogel core when stained with 6 μM calcein^AM^ (green) and 4 μM ethidium homodimer-I (red) on day 24 of culture. Live cells are represented in green and yellow arrows indicate the direction of the migration of cells from the surrounding cartilage to the hydrogel core. Yellow dotted line shows the boundary between the hydrogel core and the surrounding cartilage. Transmitted light micrographs shows the phase-contrast image of hydrogel-cartilage constructs. Scale bar represents 100 μm

Increasing the availability of cells for migration at the wound edge has been shown to improve integration of cartilage surfaces [21, 22]. Therefore, cellular migration under cLIUS in the presence of IL-6 or TNFα was studied in 2D and 3D formats. Migration of cells from the surrounding cartilage tissue into the cell-free central hydrogel core in cartilage-hydrogel constructs was visualized by live (green)/dead (red) staining and presented in Fig. 7 under different treatment conditions. On day 24, an enhanced cell migration to the hydrogel core was noted in all constructs that received cLIUS stimulation irrespective of IL-6 or TNFα treatment (Fig. 7d-f).

**Fig. 8:**
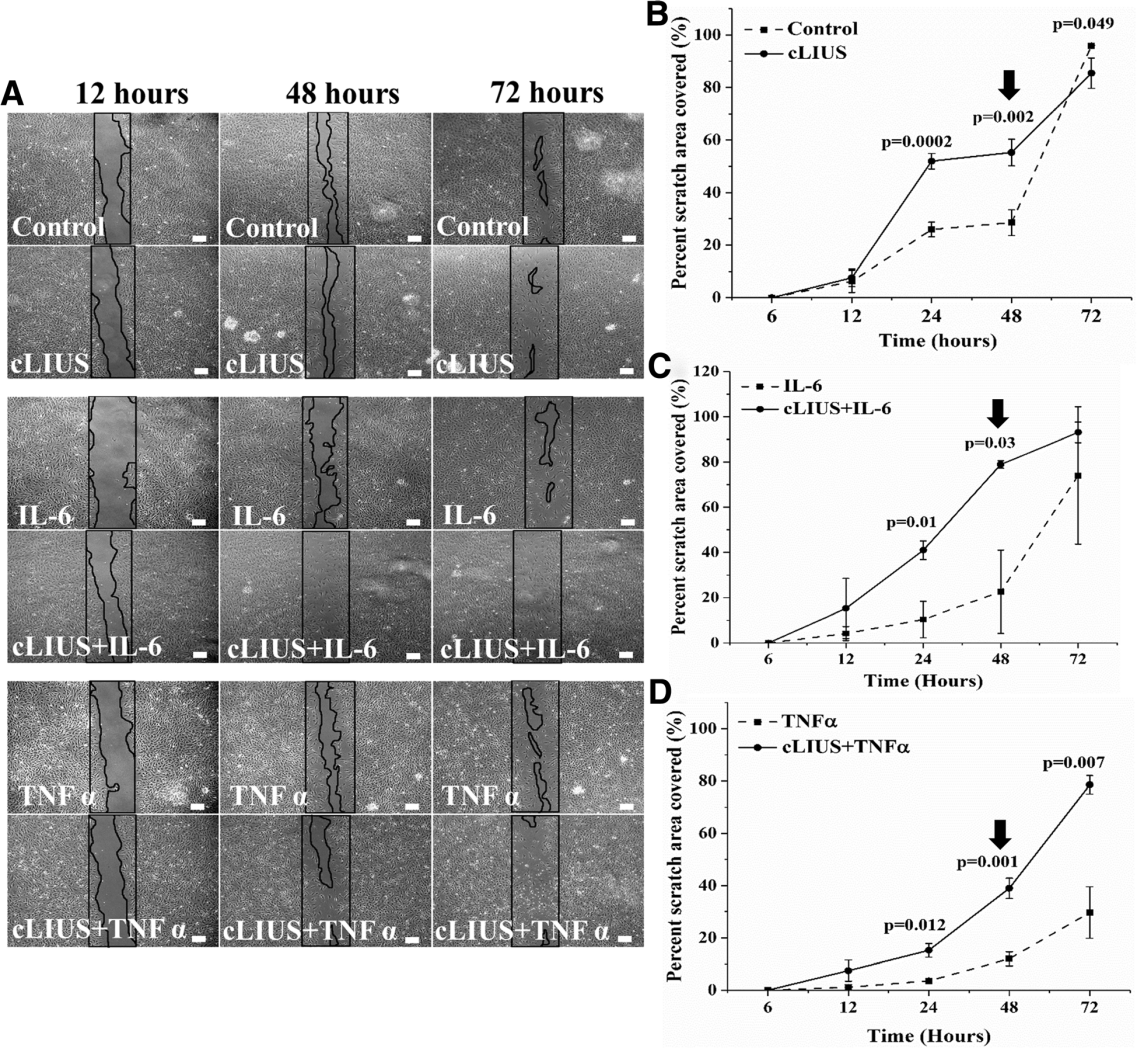
Migration of chondrocytes under cLIUS and cytokines as measured in scratch assays. **a** The figure depicts phase contrast micrographs of the scratch area of chondrocytes at 12, 48 and 72 h (*n* = 3 scratch areas in 3 separate wells, 5× magnification; scale bar = 100 μm) in the presence or absence of IL-6 or TNFα under cLIUS at 14 kPa (5 MHz, 2.5 Vpp) for 20 min. Non-cLIUS-stimulated chondrocytes served as control (*n* = 3 scratch areas in 3 separate wells). Percentage scratch area closed by migrating chondrocytes at 6, 12, 24, 48 and 72 h normalized to the wound area at 0 h under no cytokine (**b**), IL-6 (**c**) and TNFα (**d**) were quantified by ImageJ software. Data represented as mean ± 95% confidence interval

To better explain the results observed in 3D format in Fig. 7, a 2D scratch was employed to study the migration of adult chondrocytes under cLIUS stimulation in the presence or absence of IL-6 or TNFα and is shown in Fig. 8a. After 48 h of cLIUS stimulation, the migration of chondrocytes was significantly increased in the presence or absence of IL-6 or TNFα, as evidenced by 55.27 ± 5.07%, 78.94 ± 1.65%, and 38.96 ± 3.89% coverage of the scratch area in cLIUS, cLIUS+IL-6, and cLIUS+TNFα samples, respectively (indicated by black arrows in Fig. 8b-d). In contrast, the scratch area covered in non-cLIUS stimulated, and chondrocytes treated with IL-6 or TNFα alone was significantly reduced to 28.53 ± 4.94%, 22.62 ± 18.39% and 11.99 ± 2.67% respectively.

## Discussion

Cartilage repair protocols that promote integration with the native tissue while mitigating the catabolic effects of a pro-inflammatory environment and maintaining the cartilage phenotype are of interest. The negative effects of catabolic cytokines on cartilage have been well documented [23], however, to our knowledge studies demonstrating repair of cartilage fissures in a catabolic environment, albeit in vitro, has not been reported. Elevated levels of IL-6 and TNFα in the injured cartilage were implicated in the IL-6 and TNFα-mediated cartilage degradation [7]. Moderate dynamic compression and pLIUS was previously shown to mitigate the cytokine-induced catabolic effects [11, 17] in cartilage explants via the suppression of NF-κB. Our study combines an established in vitro cartilage integration model with catabolic conditions imparted by IL-6 or TNFα and demonstrates that when compared to non-cLIUS-stimulated explants: (1) chondral fissures were repaired expeditiously by cLIUS at 5 MHz in the absence of cytokines; (2) the expeditious repair of chondral fissures by cLIUS at 5 MHz was further achieved in the presence of IL-6 and TNFα; (3) IL-6 and TNFα-induced catabolic effects on cartilage was rescued by cLIUS. In explants exposed to cLIUS applied at the non-resonant frequency of 2 MHz, the chondral fissures remained open and the percent apposition was not significantly different from non-cLIUS-stimulated controls.

Most pharmacological interventions that employ anti-cytokine agents such as receptor antagonists for IL-1β, IL-6 or TNFα [24–26] and chondroprotective drugs [27, 28], are target-specific, abrogating the effect of a particular cytokine [7, 8]. However, targeting an individual cytokine may not be sufficiently effective in reducing the overall inflammatory response. Further, toxicity and adverse side-effects associated with the administration of anti-inflammatory agents hinder their long-term clinical usage [10]. Thus, a treatment modality that attenuates the effects of potent pro-inflammatory cytokines is required. The current study demonstrated the ability of cLIUS in suppressing the effects of two potent catabolic cytokines namely IL-6, and TNFα.

The expression of catabolic enzymes MMPs and ADAMTs that degrade collagens and aggrecanases respectively are elevated in injured cartilage [7, 29], and the catabolic response upon injury is exacerbated by the synergistic effects conferred by the pro-inflammatory cytokines including IL-6 and TNFα [9, 29]. In an environment of accelerated matrix degradation, the anabolic response to synthesize matrix falls behind [30, 31] and is often suppressed upon pathological progression. Thus, increased matrix turnover to overcome catabolic effects in injured cartilage is desirable. Anti-inflammatory therapeutic agents, while noted to rescue cartilage from proteoglycan loss under inflammation, do not have an effect on anabolic gene expression [32, 33]. In the present study, significant upregulation in the anabolic response of chondrocytes as evidenced by 5.85-fold increases in collagen II (Fig. 6d) and 3.15-fold increase in TIMP1 gene expression under cLIUS (Fig. 6e) accompanied the downregulation of catabolic genes, MMP13 and ADAMTS4 upon cLIUS stimulation in the presence of IL-6 or TNFα (Fig. 6a, b). Additionally, the expression of anabolic genes observed under cLIUS was consistent with the higher intensity of collagen and proteoglycan staining noted in explants (Fig. 5d-f, j-l). Collectively, cLIUS elicited a coordinated response that promoted cartilaginous matrix synthesis while simultaneously protecting the matrix from IL-6 or TNFα –induced degradation.

The key transcription factor, NF-κB, regulates inflammation by inducing the expression of pro-inflammatory cytokines and mediators, including TNFα which in turn potentiates activation of NF-κB [34, 35]. The inhibition of TNFα-induced upregulation of NF-κB by cLIUS in the present study (Fig. 6c) demonstrated the effective modulation of the NF-κB inflammation pathway in chondrocytes by cLIUS. Evidence of complete abrogation of IL-1β –induced NF-κB activation upon LIUS stimulation in a previous study [11] lends further credence to the overall anti-inflammatory effect of LIUS. Differently from the NF-κB pathway, IL-6 induces the expression of catabolic genes via the STAT3 pathway [36]. Therefore, the expression of NF-κB remained unaffected by IL-6 insult in the present study. However, elevated levels of TIMP1 expression may have rescued the IL-6-induced catabolic effects when exposed to cLIUS (Fig. 6e).

In the local milieu of the inflamed joint, an increase in the antagonistic factors that impede the migration of cells to the injured site is counterproductive to integrative repair strategies [21, 22, 37, 38]. Among the plethora of chemokines released upon inflammation; some invoke recruitment of resident and progenitor cells to initiate repair while others inhibit migration of cells. Notably, TNFα has been implicated in impeding cell migration [39] while IL-6 is known to support migration [40]. Both in the presence and absence of IL-6 or TNFα, an expeditious repair of chondral fissures noted under cLIUS, was attributed to the enhanced migration of cells.

Elongated cells were observed in TNFα-treated cartilage-hydrogel constructs (Fig. 7c, f). The cause of altered cell morphology observed is uncertain. Decreased hydrogel stiffness at lower concentrations of agarose (~ 1% agarose) was shown to affect cell shape [41]. Also, progenitor cells often display an elongated cell morphology [42] and are reported to possess greater migratory potential [43–45]. Thus, the modified cellular morphology observed could be attributed to a combination of factors, including the biophysical properties of the hydrogel and the nature of the migrating cells. Therefore, further investigations centering on the identity of migrating cells, morphological response to external physical and chemical forces including cLIUS, cytokine and/or biomaterial will be undertaken in the future. Therapeutic doses of cLIUS below 500 mW/cm^2^ do not have an adverse effect on bone growth [46]. As the cLIUS regimen employed in this study was less than < 20 mW/cm^2^, the impact of cLIUS on the bone was not investigated. However, the impact of cLIUS on both the bone and cartilage will be studied in future in vivo studies. Further, the thermal effects of cLIUS on cartilage was also not investigated in the current study as cLIUS intensity less than 20 mW/cm^2^ was not shown to elicit any thermal effects [15].

## Conclusions

Synergistic rehabilitative regeneration methods that overcome the adverse catabolic effects of the pro-inflammatory joint environment while simultaneously promoting chondro-regeneration are paramount to achieving joint restoration in the long-term. Taken together [11], results demonstrated the potential of cLIUS as a pro-anabolic, anti-catabolic and chondroprotective therapy for applications in rehabilitative regeneration programs for cartilage repair. The translation of promising in-vitro finding regarding cLIUS necessitates an understanding of the propagation of cLIUS through the joint space, an ongoing study in our laboratory.

## Additional file

Additional file 1: **Figure S1**. Alcian Blue staining of osteochondral explants under cLIUS at 2 MHz. Incised osteochondral explants were exposed to cLIUS at a non-resonant frequency of 2 MHz at 14 kPa (6 Vpp), 20 min/application, 4 applications/day for a period of 14 days in culture (*n* = 6). Non-cLIUS-stimulated explants served as controls (*n* = 6). Explants were fixed in 10% neutral buffered formalin and embedded in paraffin. Figure shows 4 μm sections of osteochondral explants at the interfacial region stained with alcian blue (pH 1) after 14 days in culture at 20× magnification. Scale bar represents 100 μm. Inserts depict the whole section imaged at 2× magnification. (TIF 9326 kb)

## Abbreviations

ACI: Autologous chondrocyte implantation
ADAMTS: A disintegrin and metalloproteinase with thrombospondin motifs
ANOVA: analysis of variance
cLIUS: Continuous low-intensity ultrasound
DMEM: Dulbecco’s modified eagle medium
FBS: Fetal bovine serum
GAG: Glycosaminoglycan
IL: Interleukin
LIUS: low-intensity ultrasound
MMP: Matrix metallopeptidase
OA: Osteoarthritis
pLIUS: Pulsed low-intensity ultrasound
TCP: Tissue culture plate
TIMP: Tissue inhibitor of metallopeptidase
TNF: Tumor necrosis factor
