# Supplementary figures and images for: Continuous low-intensity ultrasound attenuates IL-6 and TNFα-induced catabolic effects and repairs chondral fissures in bovine osteochondral explants
Source: BMC Musculoskelet Disord. 2019 May 4;20:193. doi: 10.1186/s12891-019-2566-4 (PMC6499975; doi:10.1186/s12891-019-2566-4)

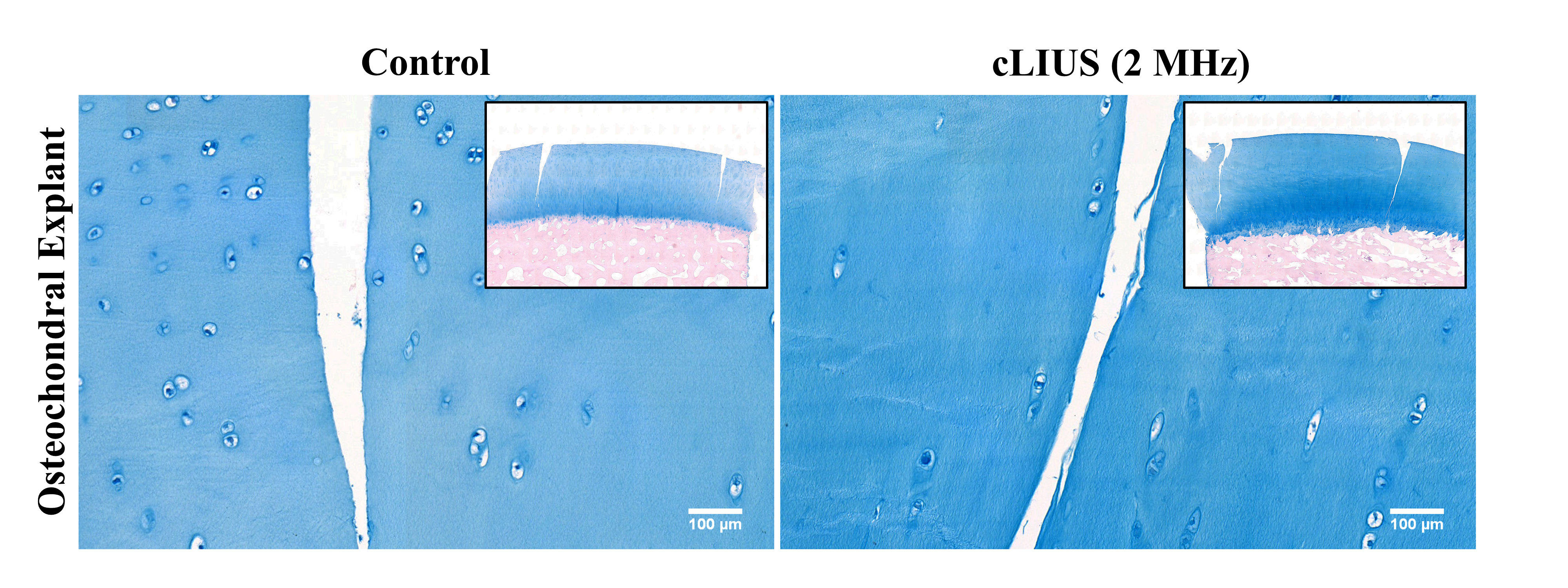

Supplement: Supplementary file 1 — Figure S1. Alcian Blue staining of osteochondral explants under cLIUS at 2 MHz. Incised osteochondral explants were exposed to cLIUS at a non-resonant frequency of 2 MHz at 14 kPa (6 Vpp), 20 min/application, 4 applications/day for a period of 14 days in culture (n = 6). Non-cLIUS-stimulated explants served as controls (n = 6). Explants were fixed in 10% neutral buffered formalin and embedded in paraffin. Figure shows 4 μm sections of osteochondral explants at the interfacial region stained with alcian blue (pH 1) after 14 days in culture at 20× magnification. Scale bar represents 100 μm. Inserts depict the whole section imaged at 2× magnification. (TIF 9326 kb) [file 12891_2019_2566_MOESM1_ESM.tif]
